# From molecular to immuno-pharmacology: Schmiedeberg Medal for Klaus Resch

**DOI:** 10.1007/s00210-025-04129-4

**Published:** 2025-04-15

**Authors:** Michael U. Martin, Detlef Neumann, Michael Kracht

**Affiliations:** 1 Springe, 31832 Germany; 2 Institute of Pharmacology, Hannover Medical School, Hannover, 30625 Germany; 3 Rudolf Buchheim Institute of Pharmacology, Justus Liebig University Giessen, Giessen, 35392 Germany


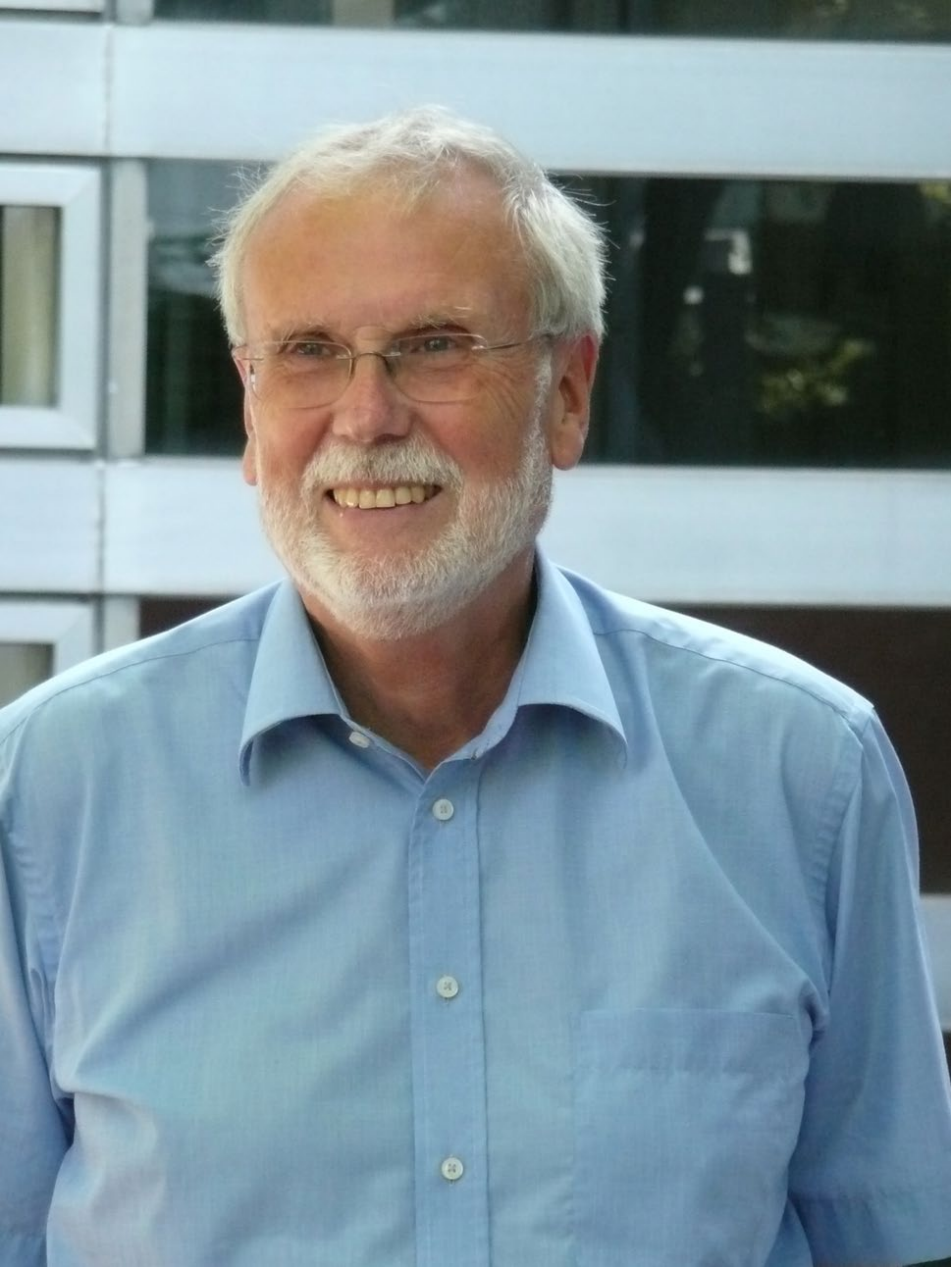


The German Society for Experimental and Clinical Pharmacology and Toxicology (DGPT) has awarded the Schmiedeberg Medal, the DGPT’s highest honour, to Professor Klaus Resch from Hannover. The medal is given to scientists who have made outstanding contributions to pharmacology and toxicology over decades. The medal was presented to Klaus Resch by Roland Seifert, President of the German Society for Pharmacology (DGP), on the occasion of the 10th German Pharm-Tox Summit, which is also the 91st Annual Meeting of the German Society for Experimental and Clinical Pharmacology and Toxicology (DGPT, on March 26, 2025.

Born in Berlin in 1941, Klaus Resch initially studied physics for two semesters before switching to human medicine, which he pursued in Berlin, Munich, and Freiburg, earning his state examination in 1966. He completed his doctorate at the Biochemical Institute in Freiburg in 1967. Captivated by the then-nascent field of immunology, he joined the Max Planck Institute for Immunobiology as a scientific assistant under its director Herbert Fischer. By 1974, he had habilitated in immunobiology, and from 1974 to 1981, worked in Heidelberg as a senior assistant and later as a Heisenberg fellow of the German Research Foundation (DFG) at the German Cancer Research Center. His Heidelberg years were interspersed by a stay as a visiting scientist at the NIH in Bethesda, USA. In 1981, he assumed the C4 professorship in Molecular Pharmacology at the Hannover Medical School’s Center for Pharmacology and Toxicology.

In March 2025, the web portal ScholarGPS, which hosts data on 30 million academic, industrial, and government scholars from 120,000 institutions in over 200 countries, is listing 292 publications authored by Klaus Resch with 12,406 citations and an h-index of 60 (ScholarGPS. 2025).

The earliest studies published by Klaus Resch trace back to 1971, when he and his colleagues showed that rabbit lymphocytes activated by phytohemagglutinin (PHA) or goat antiserum against rabbit IgG (GARIG serum) exhibited cytotoxicity, with differences in the kinetics of target cell destruction. While PHA activation resulted in immediate, linear lysis, GARIG serum induced a delayed lysis; and combining both stimulants significantly enhanced target cell destruction beyond their individual effects (Fischer et al. 1971; Resch et al. 1971; Resch and Fischer 1971). In the following years, he continued to investigate the connection between lymphocyte activation, cytotoxicity and the role of membrane associated changes in lipids and other molecules in these processes (Resch 1976).

In 1977, he published a study in nature challenging the current concepts of the signaling cascades mediating lymphocyte activation at the plasma membrane and subsequent mitosis and proliferation during immune cell activation, by showing that two microtubule active drugs, colchicine and vinblastine, failed to affect events which occur early in stimulated lymphocytes, such as increased synthesis of RNA and the protein lymphotoxin as well as the elevated turnover of membrane phospholipids (Resch et al. 1977).

Around this time, he also widened his investigations to include innate immune cell activation. He discovered, together with Diethard Gemsa, that under normal conditions, phagocytosis, a fundamental property of macrophages, increased the sensitivity to the proinflammatory prostaglandin PGE_1_ and its effects on macrophage function. However, excessive amounts of PGE_1_ antagonized this process and promoted uncontrolled inflammation (Gemsa et al. 1978). Several further studies unraveled the activation mechanisms and responses of lymphocytes and macrophages to pan-mitogenic stimuli such as concavalin A and calcium-elevating ionophores such as A23187 (Northoff et al. 1978; Gemsa et al. 1979).

By then, Marta Samel joined his lab in Heidelberg and later on moved with him to Hannover, and they started to develop biochemical approaches to study changes in membrane lipids during early lymphocyte activation (Szamel et al. 1981; Szamel and Resch 1981; Resch et al. 1981; Goppelt and Resch 1984).

Together with new team members Margarte Goppelt and Volkhard Kaever, they found that the lymphocyte plasma membrane contained high specific activities of both adenylate cyclase and guanylate cyclase, suggesting that these enzymes could be a source for cyclic nucleotides and contribute to lymphocyte growth and differentiation (Kaever et al. 1984).

The focus now also started to shift more towards immunopharmacology by showing together with Michael Stoeck, David Lovett, and Michael Martin that some of the earliest events in the course of lymphocyte activation, the enhanced incorporation of unsaturated fatty acids and phospholipids into the plasma membrane of activated lymphocytes was attenuated by cyclosporine A, revealing a new mode of action for this novel and very potent immunosuppressive drug (Stoeck et al. 1985; Szamel et al. 1985).

In the mid-80s, Klaus Resch and colleagues started also to work on activation mechanisms and biological functions of the novel cytokine interleukin-1 (IL-1) and characterized, together with Heinfried Radeke, Beate Schwinzer, and Peter Uciechowski, its actions on macrophages and glomerular mesangial cells, as an innovative model of tissue-resident cells with immune functions (Lovett et al. 1987, 1986; Radeke et al. 1994).

A seminal finding was the discovery, by protein-based biochemical methods established by Michael Martin, that IL-1 induced the specific phosphorylation of a 41 kDa protein resident within the plasma membrane, offering a new molecular explanation how this cytokine would transduce signals from its receptor into the cytoplasm of the responding cell (Martin et al. 1986, 1989).

This work inspired several follow-up studies and collaborations spear-headed by Michael Martin and Klaus Resch together with Heinfried Radeke, Roswitha Kroggel, and Brigitte Brigelius-Flohe´ on the IL-1 receptor complex and early IL-1 signal transmission (Kroggel et al. 1988; Radeke et al. 1991; Brigelius-Flohe et al. 1993; Martin and Resch 1988). These efforts eventually resulted in the characterization of an IL-1 receptor associated protein kinase activity, which was probably one of the first reports on the later identified IRAK- 1 (Martin et al. 1994). Together with new institute members Holger Wesche and Detlef Neumann, they then identified the IL-1 receptor accessory protein (IL-1RAcP, now called IL-1R3) as the mandatory second co-receptor chain of the IL-1 receptor heterodimer, and elucidated the mechanism of activation of IRAK-1. The work on IL-1 and IRAKs has been highly cited and continued until Klaus Resch’s retirement (Wesche et al. 1996, 1997, 1998; Neumann et al. 2007, 2008).

In the 1990s, the institute became more diverse, with new members and groups being recruited. Helmut Holtmann and Klaus Resch started to work on tumor necrosis factor (TNF)α receptors and downstream mechanisms (Konig et al. 1991; Holtmann et al. 1991; Holtmann and Resch 1995). Michael Kracht, Marta Szamel, and Klaus Resch unraveled signaling mechanisms of IL-2 in T lymphocytes (Szamel et al. 1990; Kracht et al. 1993). Thereafter, Helmut Holtmann and Michael Kracht initiated their own groups under guidance of Klaus Resch and combined efforts to work together with Reinhard Winzen on stress-activated protein kinases and how they regulated cytokine-mediated gene expression with a focus on transcriptional and post-transcriptional control. This included the establishment of technologies to assess the transcriptome-wide gene expression response of cytokine-activated cells by microarrays (Krause et al. 1998; Buss et al. 2004; Holtmann et al. 1999, 2001; Winzen et al. 1999; Bollig et al. 2002; Gowrishankar et al. 2005; Wolter et al. 2008).

These few selected examples of important scientific contributions demonstrate that Klaus Resch recognized the relevance of the field of immunopharmacology, i.e., the need to combine the fields of immunology and classical pharmacology, at a very early stage. As described above, he was not only an active part of the international research community for decades, but he has also promoted immunopharmacology nationally and at the Hannover Medical School at various levels because he was firmly convinced of its importance and translational potential.

When Klaus Resch began his scientific career, many of the basic principles of modern immunopharmaceuticals were still unknown and targeted therapies such as the blockade of IL-1, TNFα, or IL-17 in diseases such as rheumatoid arthritis, psoriasis, or chronic inflammatory bowel disease remained a vision.

It will therefore fulfil him with satisfaction that a large number of new drugs, such as monoclonal antibodies against individual cytokines, or “multi” cytokine blockers such as janus kinase inhibitors, are now available as effective targeted therapies for the most important autoimmune diseases. As always, the journey is not over and it is conceivable that one day cell-based therapy with modulated T cells (such as chimeric antigen receptor, CAR T cells) or individualized mRNA vaccination will be part of a physician’s standard pharmacotherapeutic repertoire.

Klaus Resch’s scientific career is therefore an example of the importance to focus, on the one hand, on a topic and, on the other hand, to remain constantly interested in new concepts and developments.

While his primary research interest was lymphocyte activation, focusing on the role of the plasma membrane and its phospholipids, he quickly expanded to macrophage activation, emphasizing prostaglandins and leukotrienes. Always open to new ideas, Klaus Resch actively contributed to two primary research areas at the Hannover Medical School: organ transplantation and inflammation. Early on, the molecular mechanisms of immunosuppressants, initially cyclosporine A and later other drugs, were explored in the department of Molecular Pharmacology. His institute also established a therapeutic drug monitoring service for immunosuppressive drugs, analyzing samples from patients nationwide. The focus on inflammation research naturally complemented existing projects, investigating complex inflammation processes using cell biology and biochemical methods.

In this regard, Klaus Resch’s work has always seamlessly bridged pharmacology and immunology into immunopharmacology, demonstrated by around 300 original and review articles and several textbooks on pharmacology, immunology, and immunopharmacology. Noteworthy, his engagement extended beyond his retirement, influencing his coworkers, who now hold responsible positions elsewhere, and guiding research on inflammation and immunosuppressants.

During his farewell in November 2008, Prof. Bitter-Suermann, the former president of the Hannover Medical School, described him as an “influential special researcher,” highlighting his significant role in establishing and organizing several collaborative research centers at the Hannover Medical School. He co-founded the highly successful Collaborative Research Center 244 on chronic inflammation, which shaped the Hannover Medical School’s research landscape for 15 years. Klaus Resch’s long-standing commitment secured vital research funding and provided an excellent environment for young scientists to present and realize their first projects.

University research inevitably involves university politics, which Klaus Resch understood early on. He actively engaged in the Hannover Medical School’s various roles, including serving as a Senate member from 1989 to 2003 and representing the Medical School’s interests as Senate speaker. His support for biochemistry and the biochemistry program at the Hannover Medical School, which faced difficult times, was particularly noteworthy. Many outside Hannover remember him as a busy reviewer for the DFG, whose critical yet fair assessments helped to establish numerous Collaborative Research Centers across Germany. Nationally, he was active in immunology and pharmacology societies, often serving on their boards. At DGPT conferences, Klaus Resch significantly contributed to the establishment of immunopharmacological sessions, highlighting his pioneering role in the field.

Despite his numerous responsibilities, Klaus Resch has managed to contribute successfully to the Hannover Medical Orchestra, a feat that remains a mystery not only to his former students but also to his wife Silke and their two children.

Klaus Resch joined the Hannover Medical School in 1981 as an enthusiastic young Professor of Molecular Pharmacology. Despite officially retiring in 2008, he has continued his involvement, regularly delivering highly regarded pharmacology lectures to the students until the outbreak of the COVID-19 pandemic. He has consistently prioritized excellent teaching, modern practical education, and highly competitive basic research, which have been his main objectives as a university lecturer. Many cohorts of students, especially those in human medicine and biochemistry, owe him their excellent introduction into pharmacology and immunology.

Klaus Resch and his team have facilitated access to immunopharmacology for countless doctoral students in medicine, biology, and particularly biochemistry. One of his most outstanding achievements over decades was creating an exciting environment where scientific exchange occurred among nearly equals. His assertion that “he was always a beginner in a new research area” quickly dissolved barriers between the “professor up there” and young beginners “down here”, fostering an unusually fruitful scientific exchange. His willingness to engage in new projects enabled various research groups under his leadership to continually develop and successfully implement new and competitive research focuses.

In a sense, Klaus Resch was never the typical pharmacologist; he showed a keen interest in immunology even during his student years. Early in his career, he preferred using cell biology and biochemistry methods, later expanding to molecular biology. In summary, he can best be described as an immunopharmacologist who masterfully connects the worlds of immunology and pharmacology.

It is a great honour for the DGPT to award the Schmiedeberg Medal to Professor Klaus Resch.
